# The complete mitochondrial genome and phylogenetic analysis of the deep-sea limpet *Bathyacmaea lactea*

**DOI:** 10.1080/23802359.2021.1923422

**Published:** 2021-06-23

**Authors:** Yaran Liu, Yuli Li, Minxiao Wang, Zhenmin Bao, Shi Wang

**Affiliations:** a Key Laboratory of Marine Genetics and Breeding (Ministry of Education), Ocean University of China, Qingdao, China; b Laboratory for Marine Biology and Biotechnology, Pilot National Laboratory for Marine Science and Technology, Qingdao, China; c Key Laboratory of Marine Ecology and Environmental Sciences and Deep Sea Research Center, Institute of Oceanology, Chinese Academy of Sciences, Qingdao, China; d Laboratory for Marine Fisheries Science and Food Production Processes, Pilot Qingdao National Laboratory for Marine Science and Technology, Qingdao, China

**Keywords:** *Bathyacmaea lactea*, mitochondrial genome, phylogenetic analysis

## Abstract

The deep-sea limpet *Bathyacmaea lactea*, inhibited in cold seeps of the South China Sea, belongs to the most primitive gastropod groups, the Patellogastropoda (true limpets). Here, we report the complete mitochondrial genome of *B. lactea*, which is 18,446 bp in length and contains 13 protein-coding genes, 2 rRNAs, and 22 tRNA genes. The mitochondrial genome of *B. lactea* enriches the molecular resources for further understanding deep-sea molluskan adaptation and Gastropoda evolution.

*Bathyacmaea lactea* distributes in deep sea areas in the Western Pacific (Zhang et al. 2016), which belongs to the family Acmaeidae. The deep-sea ecosystem is largely unexplored and the omics resources of deep-sea animals are limited. Mitochondrial genes have been extensively used for studying population structure and phylogenetic relationships at various taxonomic levels (Saccone et al. 1999; Boore et al. 2005). The number of reported deep-sea limpets was 23 according to the World Register of Deep-Sea species (WoRDSS) website, and six mitochondrial genomes of deep-sea limpets have been reported (Uribe et al. 2019; Glover et al. 2021). In this study, we present the complete mitochondrial genome of the limpet *B. lactea* collected from cold seeps in South China Sea, which will provide as useful molecular resources for further understanding of Gastropoda phylogeny and evolution.

The *B. lactea* sample was collected from cold seeps in the South China Sea (22° 06′ N, 119° 17′ E, depth 1168 m) using the remotely operated vehicle (ROV) of *Kexue*. Genomic DNA was extracted from muscle tissue of the limpet by using CTAB method (Winnepenninckx et al. 1993). The DNA sample, as well as the shell and left tissue of the individual were deposited in −80 °C storage freezer which in the Key Laboratory of Marine Genetics and Breeding (Ministry of Education), Ocean University of China (http://mgbkl.ouc.edu.cn/, Specimen code: OUC-MGB-2018-BL-10, Yuli Li, liyuli@ouc.edu.cn) under the voucher number BL201810. Whole-genome sequencing of the sample was performed using the Illumina HiSeq X Ten platform. The mitogenome of *B. lactea* was *de novo* assembled by using NOVOPlasty (Dierckxsens et al. 2017), and then annotated by the MITOS web server (Bernt et al. 2013). Finally, the mitogenome was manually corrected.

The complete mitogenome of *B. lactea* was 18,446 bp in length and encoded 13 protein-coding genes (PCGs), 2 ribosomal RNA genes (rRNAs) and 22 transfer RNA genes (tRNAs). The mitogenome sequence was submitted to the GenBank under the accession number of MW309841. The order of 13 PCGs and 2 rRNA genes of *B. lactea* is consistent with the hypothetical ancestral gastropod (Uribe et al. 2019). In 13 conserved PCGs, nine of them start with ATG including cox1, cox2, cox3, cob, nad1, nad3, nad5 and nad4l, while the rest PCGs start with ATT. For the stop codon, the atp6 and cox3 gene stops with TAG, while the other PCGs terminates with TAA. The *B. lactea* mitogenome is AT rich with a base composition of 32.31% A, 37.53% T, 9.93% C and 20.23% T, which is similar to other gastropod species (Schultz et al. 2018). The small subunit ribosomal RNA (12S rRNA) and large subunit ribosomal RNA (16S rRNA) were annotated with sizes of 887 bp and 1,346 bp, respectively. In addition, the length of 22 tRNA genes varied from 55 to 71 bp, and the order of them was tRNA^Asn^, tRNA^Asp^, tRNA^Thr^, tRNA^Gly^, tRNA^Lys^, tRNA^Ile^, tRNA^Ser1^, tRNA^His^, tRNA^Gln^, tRNA^Ser2^, tRNA^Pro^, tRNA^Leu2^, tRNA^Leu1^, tRNA^Val^, tRNA^Tyr^, tRNA^Met^, tRNA^Phe^, tRNA^Trp^, tRNA^Cys^, tRNA^Glu^, tRNA^Ala^ and tRNA^Arg^.

The phylogenetic tree was constructed based on 13 PCGs of the *B. lactea* and other 15 gastropods from six main lineages, including Patellogastropoda, Neomphalina, Vetigastropoda, Neritimorpha, Caenogastropoda and Cocculiniformia. And the *Octopus bimaculatus* and *Octopus vulgaris* were used as outgroup (Figure 1). Gene sequences were downloaded from NCBI database and were aligned by using MAFFT software (Katoh et al. 2019), then a phylogenic tree was constructed using the Maximum-likelihood (ML) method with LG + G model by using RAxML software (Stamatakis 2014) and visualized by iTOL web server (Letunic and Bork 2019). As the tree indicated, *B. lactea* was closely related to the species *Bathyacmaea nipponica*, both of which belong to the earliest-branching group of Petellogastropoda, which is the sister taxon to all other gastropod taxa (Kocot et al. 2011). The evolution relationships between the six gastropod lineages are consistent with previous studies (Lee et al. 2019; Sun et al. 2019).

**Figure 1.**
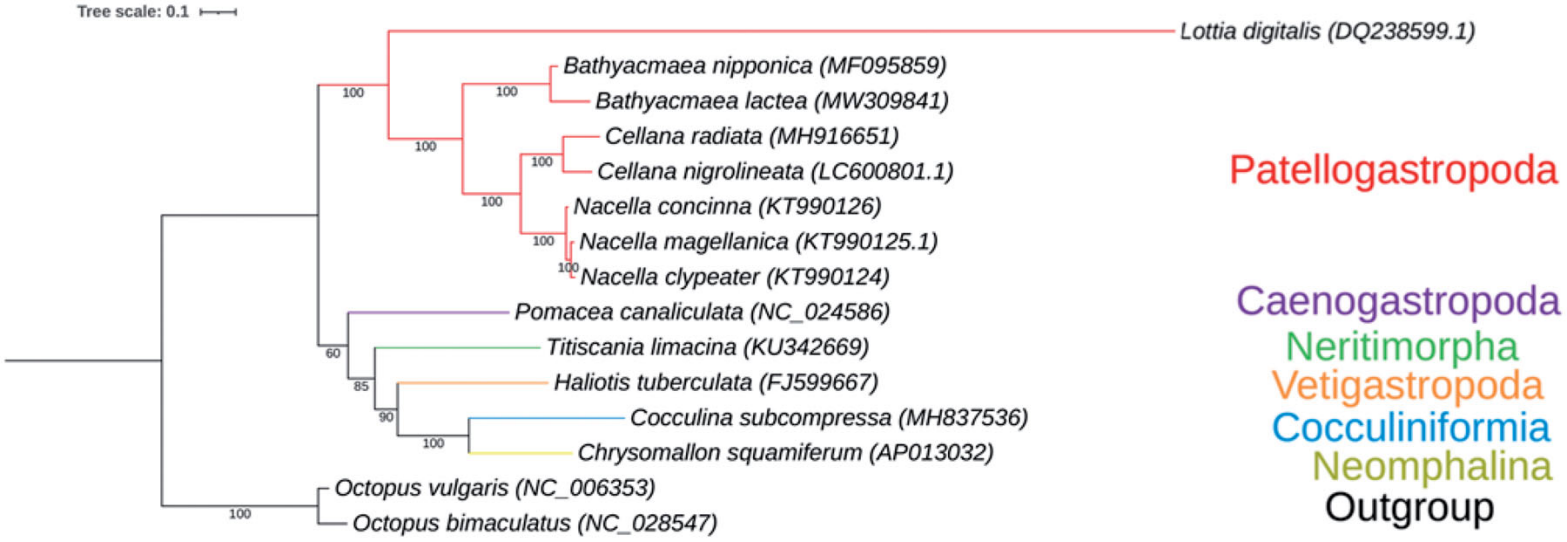
The Maximum-likelihood phylogenetic tree for *B. lactea* and the other Gastropoda species based on 13 protein-coding genes, and *B. lactea* is placed within Patellogastropoda.

## Data Availability

The genome sequence data that support the findings of this study are openly available in GenBank of NCBI at https://www.ncbi.nlm.nih.gov under the accession number MW309841. The associated BioProject, SRA and Bio-Sample numbers are PRJNA713489, SRR13932351 and SAMN18252891, respectively.
